# RfpA, RfpB, and RfpC are the Master Control Elements of Far-Red Light Photoacclimation (FaRLiP)

**DOI:** 10.3389/fmicb.2015.01303

**Published:** 2015-11-25

**Authors:** Chi Zhao, Fei Gan, Gaozhong Shen, Donald A. Bryant

**Affiliations:** ^1^Department of Biochemistry and Molecular Biology, The Pennsylvania State University University Park, PA, USA; ^2^Department of Chemistry and Biochemistry, Montana State University Bozeman, MT, USA

**Keywords:** photosynthesis, photosystem I, photosystem II, phycobilisome, chlorophyll *f*, chlorophyll *d*, *Chlorogloeopsis fritschii* PCC 9212, *Chroococcidiopsis thermalis* PCC 7203

## Abstract

Terrestrial cyanobacteria often occur in niches that are strongly enriched in far-red light (FRL; λ > 700 nm). Some cyanobacteria exhibit a complex and extensive photoacclimation response, known as FRL photoacclimation (FaRLiP). During the FaRLiP response, specialized paralogous proteins replace 17 core subunits of the three major photosynthetic complexes: Photosystem (PS) I, PS II, and the phycobilisome. Additionally, the cells synthesize both chlorophyll (Chl) *f* and Chl *d*. Using biparental mating from *Escherichia coli*, we constructed null mutants of three genes, *rfpA, rfpB*, and *rfpC*, in the cyanobacteria *Chlorogloeopsis fritschii* PCC 9212 and *Chroococcidiopsis thermalis* PCC 7203. The resulting mutants were no longer able to modify their photosynthetic apparatus to absorb FRL, were no longer able to synthesize Chl *f*, inappropriately synthesized Chl *d* in white light, and were unable to transcribe genes of the FaRLiP gene cluster. We conclude that RfpA, RfpB, and RfpC constitute a FRL-activated signal transduction cascade that is the master control switch for the FaRLiP response. FRL is proposed to activate (or inactivate) the histidine kinase activity of RfpA, which leads to formation of the active state of RfpB, the key response regulator and transcription activator. RfpC may act as a phosphate shuttle between RfpA and RfpB. Our results show that reverse genetics via conjugation will be a powerful approach in detailed studies of the FaRLiP response.

## Introduction

Terrestrial cyanobacteria often occur in niches that are strongly enriched in far-red light (FRL; λ > 700 nm). Soil and fresh-water cyanobacteria encounter FRL because it penetrates more deeply into soils and because plant canopies strongly enrich for FRL because of absorption by chlorophylls (Chl) *a* and *b* (Gan and Bryant, 2015). Similar effects occur in microbial mats and sometimes in blooms, where self-shading can provide a powerful selection for alternative, light-harvesting antenna systems (Kühl and Jørgensen, 1994; Nowack et al., 2015; Olsen et al., 2015). We recently discovered two photoacclimation processes that occur in some cyanobacteria in FRL and/or low light, here denoted Far-Red Light Photoacclimation (FaRLiP) (Gan et al., 2014, 2015; Gan and Bryant, 2015) and Low Light Photoacclimation (LoLiP) (Nowack et al., 2015; Olsen et al., 2015). During the FaRLiP response, specialized paralogous proteins replace 17 core subunits of the three major photosynthetic complexes: Photosystem (PS) I, PS II, and the phycobilisome. Additionally, the cells synthesize both Chl *f* and Chl *d* (Gan et al., 2014, 2015). The net impact is that the remodeled photosynthetic apparatus confers the ability to absorb light between 700 and 800 nm, and thus cells are able to grow in FRL. The LoLiP response occurs within low-light-adapted ecotypes of *Synechococcus* spp. in microbial mats associated with Mushroom Spring in Yellowstone National Park. In this acclimation response, cells only synthesize Chl *a*, but a three-gene operon, *apcD4-apcB3-isiX*, is expressed that leads to absorption beyond 700 nm, and cells exhibit improved growth at low irradiance (Nowack et al., 2015; Olsen et al., 2015). The *apcD4-apcB3-isiX* operon does not occur in high-light-adapted ecotypes of *Synechococcus* sp. (Olsen et al., 2015), but these genes do occur sporadically in some other cyanobacteria (Gan et al., 2015).

Analyses of all publicly available cyanobacterial genomes show that 13 cyanobacteria have the gene cluster that confers the capacity for FaRLiP (Gan et al., 2014, 2015). Three other cyanobacteria have been shown to synthesize Chl *f* when grown in light conditions consistent with FaRLiP, and presumably these strains will also contain similar gene clusters (Gan and Bryant, 2015). Each FaRLiP gene cluster contains a three-gene operon, *rfpB-rfpA-rfpC*, that encodes regulatory proteins. RfpA is a red/far-red, knotless phytochrome, and these proteins form a distinctive structural family of cyanobacterial photoreceptors (Gan et al., 2014). RfpA has a histidine kinase domain and is proposed to act as the sensor kinase in a signal transduction cascade. RfpB is the response regulator and contains a winged-helix, DNA-binding domain as well as two CheY-like domains. RfpC is also a CheY-like protein and may act to transfer a phosphate from RfpA to RfpB. Although there is strong circumstantial evidence that the products of these three genes are the primary regulatory proteins of the FaRLiP response, because no genetic system had been developed in any verified FaRLiP strain, this point has not been directly demonstrated until now.

In the work reported here, we selected two common, terrestrial cyanobacteria, *Chlorogloeopsis fritschii* PCC 9212 and *Chroococcidiopsis thermalis* PCC 7203 (hereafter *Chl. fritschii* PCC 9212 and *Chr. thermalis* PCC 7203), which perform FaRLiP to demonstrate that RfpA, RfpB, and RfpC are the master control elements of the response to FRL. *Chl. fritschii* is a developmentally complex cyanobacterium belonging to the order *Stigonematales* (subsection 5, *Cyanobacteria*) (Evans et al., 1976; Hoffman and Castenholz, 2001). The type strain of *Chl. fritschii* (strain PCC 6912) was isolated by Mitra and Pandey from soil in India (Mitra and Pandey, 1967), but *Chl. fritschii* strains are also reported to occur in hot springs worldwide at temperatures up to 63–64°C. The strain used in the studies reported here, *Chl. fritschii* PCC 9212 was isolated from a hot spring in Ourense, Spain. Despite these very different habitats, the genomes of the two *Chl. fritschii* isolates, PCC 6912 and 9212, are very similar.

*Chr. thermalis* PCC 7203 was initially described as a “primitive” cyanobacterium (Friedmann et al., 1994; Friedmann and Ocampo-Friedmann, 1995), but its large genome and a complex developmental cycle involving the production of non-motile baeocytes contradicts this notion. Furthermore, phylogenetic analyses show that *Chroococcidiopsis* spp. is not a member of the order *Pleurocapsales*, but rather that its closest relatives are heterocystous cyanobacteria (*Nostocales* and *Stigonematales*) (Fewer et al., 2002). *Chroococcidiopsis* spp. are extremely desiccation resistant and are among the most resistant organisms known against ionizing radiation (Billi et al., 2001). *Chr. thermalis* PCC 7203 was isolated from soil, but members of this genus are often endoliths and/or hypoliths and are found in a wide range of extreme environments, including hot and cold deserts, soils, hot springs, and caves as well as in freshwater, marine and hypersaline conditions (Friedmann et al., 1994; Friedmann and Ocampo-Friedmann, 1995). Note that many of these environments produce light conditions enriched in FRL, and thus not surprisingly, *Chr. thermalis* PCC 7203 has a FaRLiP gene cluster (Gan et al., 2014, 2015).

In this study we show that DNA can be introduced by conjugation into *Chl. fritschii* PCC 9212 and *Chr. thermalis* PCC 7203 and that gene deletions can be obtained by double recombination without the necessity to counter-select with sucrose/SacB. Using this approach, we constructed deletion mutations for *rfpA, rfpB* and *rfpC*. The phenotypes of these mutants establish that RfpA, RfpB and RfpC form the master control switch for the FaRLiP response. These studies set the stage for more exhaustive use of reverse genetics to study the roles the paralogous FaRLiP genes in facilitating growth in FRL.

## Materials and methods

### Strains and growth conditions

The cyanobacterial strains used in this study were obtained from the Pasteur Culture Collection (http://www.pasteur.fr/pcc_cyanobacteria) (Rippka et al., 1979; Rippka, 1988). *Chl. fritschii* PCC 9212 was isolated from a hot spring near Ourense, Spain. *Chr. thermalis* PCC 7203 was isolated from soil near Greifswald, Germany. These two strains were grown in the B-HEPES growth medium (Dubbs and Bryant, 1991), a modified BG11 medium containing 1.1 g L^−1^ 4-(2-hydroxyethyl)-1-piperazineethanesulfonic acid (HEPES) (final concentration) with the pH adjusted to 8.0 with 2.0 M KOH. Warm white fluorescent lights provided continuous illumination at ~250 μmol photons m^−2^ s^−1^ (WL), and liquid cultures were sparged with 1% (v/v) CO_2_ in air. FRL was provided by LEDs (Marubeni, Santa Clara, CA) with emission centered at 720 nm (15–18 μmol photons m^−2^ s^−1^). Plastic filters, the transmittance properties of which have been previously described (Gan et al., 2014), were sometimes used to produce green light (GL), red light (RL), and FRL. Growth of cells was monitored turbidometrically at 730 nm by using a GENESYS 10 spectrophotometer (ThermoSpectronic, Rochester, NY).

### Construction of mutants and conjugation

The biparental conjugation system was kindly provided by Professor Jindong Zhao from Peking University (Zhao et al., 2005). The donor *E. coli* strain HB101 contained the conjugal plasmid pRL443 and the helper plasmid pRL623 (Elhai and Wolk, 1988; Elhai et al., 1997). To generate constructs for deletion of the *rfpA, rfpB*, or *rfpC* genes, an *ermC* cassette, which confers resistance to erythromycin, was amplified through PCR and cloned onto the cargo plasmid pRL277 using SacI and SphI restriction sites (see Table S1 for primer sequences). For each of the target genes, upstream and downstream DNA fragments with a size of ~2.5–3.0 kb were amplified by PCR using Phusion® HF DNA polymerase (New England Biolabs, Ipswich, MA, USA). The primers for DNA fragment amplification of the upstream and downstream regions of *rfpA, rfpB*, and *rfpC* genes from *Chl. fritschii* PCC 9212 and *Chr. thermalis* PCC 7203 *rfpA* and *rfpB* are listed in Table S1. Upstream and downstream DNA fragments for each target gene were cloned onto the cargo plasmid pRL277 with an interposing DNA fragment encoding *ermC* to replace the target gene through a double-crossover recombination event.

Cargo plasmids were transformed into the donor *E. coli* HB101 cells containing the conjugal plasmid pRL443 and the helper plasmid pRL623. The resulting *E. coli* HB101 strains were grown in 5–20 ml of Luria-Bertani (LB) medium supplemented with appropriate antibiotics and cultured at 37°C overnight. The *E. coli* cells were harvested by centrifugation at low speed, washed with fresh LB medium three times, and resuspended in fresh LB or B-HEPES medium (100–200 μl). Meanwhile, freshly grown wild-type *Chl. fritschii* PCC 9212 or *Chr. thermalis* PCC 7203 cells (OD_730nm_ = 0.6–1.0; 500 μl to 2.0 ml) were centrifuged at low speed, washed with fresh B-HEPES medium three times, and finally resuspended in B-HEPES medium (100–200 μl). The *E. coli* and cyanobacterial cells were gently mixed in a sterile microcentrifuge tube and incubated at 30°C under low light for 4–6 h, and the cell mixture was then spread onto a sterile nitrocellulose filter overlaid on a B-HEPES agar plate without antibiotics. The plate was incubated at 30°C under low light for another 18–36 h, and then the filter was transferred to a B-HEPES agar plate containing 20 μg erythromycin ml^−1^. Green-colored colonies appeared on the filter after 4–6 weeks; they were picked and streaked repeatedly on selective medium, and the purified transconjugant cells were ultimately transferred to liquid B-HEPES medium for analyses. Segregation of wild-type and mutant alleles of the target gene was analyzed by colony PCR and by PCR analysis of cells from liquid cultures.

### Pigment measurements and absorption and fluorescence spectroscopy

The Chl and carotenoid contents of cells were measured from pigments extracted with 100% methanol. Spectroscopic measurements were performed with a GENESYS 10 UV–Vis spectrophotometer (Thermo Scientific). The Chl *a* concentration was determined on the basis of equivalent cell concentrations as determined by equal OD_730nm_ values as described (Shen et al., 2008). To measure the absorption spectra of whole cells, cells from liquid cultures were harvested and resuspended in 10% (w/v) sucrose prepared with 50 mM HEPES buffer, pH 7.0. Homogenization with a Teflon/glass homogenizer was used to achieve a more homogenous suspension of the filamentous cells of the cyanobacterial strains employed in this study. Absorption spectra of cell cultures were measured with a UV–Vis–NIR Cary™ 14 spectrophotometer that was modified for computerized data collection and analysis by On-Line Instrument Systems, Inc. (Bogart, GA).

Fluorescence emission spectra at low temperature (77 K) were measured using an SLM Model 8000C spectrofluorometer that was modified for computerized solid-state operation by On-line Instrument Systems Inc., (Bogart, GA) (Shen et al., 2008). Wild-type and mutant cells were harvested from liquid medium and resuspended in 50 mM HEPES/NaOH pH = 7.0 buffer containing 60% (v/v) glycerol by gentle homogenization. After loading samples into the measuring tubes, cells were quickly frozen in liquid nitrogen. To measure the fluorescence emission from Chl-protein complexes, the excitation wavelength was 440 nm, which selectively excites Chls.

### Pigment extraction and HPLC analysis

For pigment extraction and HPLC analyses, cyanobacterial cells were harvested and washed once in 50 mM HEPES/NaOH buffer, pH 7.0 by centrifugation, resuspension, and subsequent centrifugation. Pigments were extracted from the cell pellet by sonication in acetone:methanol (7:2, v/v) under dim light (Graham and Bryant, 2008; Gan et al., 2014). After centrifugation to remove protein and other insoluble cell debris, pigment solutions were filtered through 0.2 μm polytetrafluoroethylene membrane syringe filters. Pigments were analyzed by reversed-phase HPLC on a 25 cm × 4.6 mm analytical Discovery C18 column (Supelco, Bellefonte, PA) using an Agilent Model 1100 HPLC system equipped with a model G1315B diode array detector. The solvent system and elution conditions were identical to those previously described (Gan et al., 2014).

### Transcription profiling by RNA-SEQ analysis

Cells grown continuously in WL were inoculated into three tubes with fresh B-HEPES medium at OD_750nm_ = 0.05 and sparged with 1% (v/v) CO_2_. When cells grew to OD_750nm_ = ~0.6, a 15-mL aliquot of culture was collected from each of the three tubes and combined. The combined culture sample was rapidly centrifuged at 4°C, and the resulting cell pellet was quickly frozen with liquid nitrogen and stored at −80°C (*T* = 0 h, WL sample). The remainder of the cultures were transferred to FRL. Aliquots of cells (45 mL in total) of cells were collected at 12, 24, and 48 h, centrifuged, and stored as described above (samples *T* = 12 h, *T* = 24 h, *T* = 48 h). One additional sample was prepared from cells grown in FRL for 2 weeks (336 h; designated as sample FR in Table S2). For RNA isolation, cell pellets were suspended in 50 mM Tris-HCl, pH 8.0. An equal volume of glass beads was mixed with the cell suspension, which was then subjected to a brief bead beating (4200 rpm for 10 s) with a mini-beadbeater (Biospec Products, Bartlesville, OK). Total RNA was extracted with phenol and further purified as described (Ludwig and Bryant, 2011) with High Pure RNA Isolation Kit (Roche, Indianapolis, IN).

Following the manufacturer's instructions, ribosomal RNA was removed using Ribo-Zero™ rRNA Removal Kit for bacteria (Epicenter, Madison WI) to obtain enriched mRNA. Construction of the cDNA library and Illumina sequencing was performed in the Genomics Core Facility, The Pennsylvania State University, University Park. Libraries were prepared from enriched mRNA using the TruSeq Stranded mRNA Sample Prep Kit (Illumina, San Diego, CA) according to the manufacturer's instructions with the exception that the poly-A selection steps at the beginning of the protocol were omitted. Samples were sequenced on an Illumina HiSeq 2500 instrument in Rapid Run mode by performing 50-nt single read sequencing according to the instructions of the manufacturer. Mapping against the *Chl. fritschii* PCC 9212 genome was performed using the BWA software package using scripts modified to accommodate the Illumina sequences (Li and Durbin, 2009). The resulting alignment files were further analyzed with self-developed scripts to extract expression levels for each gene as described previously (20). In the rRNA depleted WL sample (*T* = 0 h), 10,504,251 reads were obtained, of which 7,221,701 reads (69%) were uniquely mapped to mRNA. For the rRNA-depleted samples of cells grown in FRL 10,033,440, 9,619,193, and 13,070,108 total reads were obtained and 9,941,370 (99%), 9,545,774 (99%), and 13,034,557 (99%) reads were uniquely mapped to mRNA for *T* = 12 h, *T* = 24 h, *T* = 48 h, respectively. For the sample from cells grown in FRL for 2 weeks (sample FR), 12,171,511 total reads were obtained and 12,096,759 (99%) reads were uniquely mapped to mRNA. The RNA sequencing data were deposited in the NCBI Sequence Read Archive (SRA) under accession number SRP062739.

### Isolation of total RNA and PCR analysis

For gene expression analysis, total RNA was isolated from cells of the wild type and mutant strains grown to mid-exponential growth phase using a High Pure RNA isolation kit (Roche Diagnostics, Indianapolis, IN). To eliminate trace amounts of contaminating chromosomal DNAs, RNA samples were incubated with RNase-free DNase I (Promega, Madison, WI) for 1 h at room temperature. DNase-treated RNA samples were further purified using RNA purification cartridges. The absence of DNA in RNA samples was verified by PCR assays in which reverse transcriptase was omitted. The concentration of RNA was determined by NanoDrop measurements (Thermo Scientific, Watham, MA).

RT-PCR analysis of total RNA isolated from wild-type and mutant strains was performed using a MyTaq One-Step RT-PCR kit (Bioline USA Inc., Taunton, MA). Primers were designed to amplify specifically selected genes encoding major subunits of PS I, PS II and phycobilisomes as well as the 16S RNA gene as control (see Table S1). The amplicons from RT-PCR analysis were analyzed by agarose gel electrophoresis.

## Results

**Figure 1:**
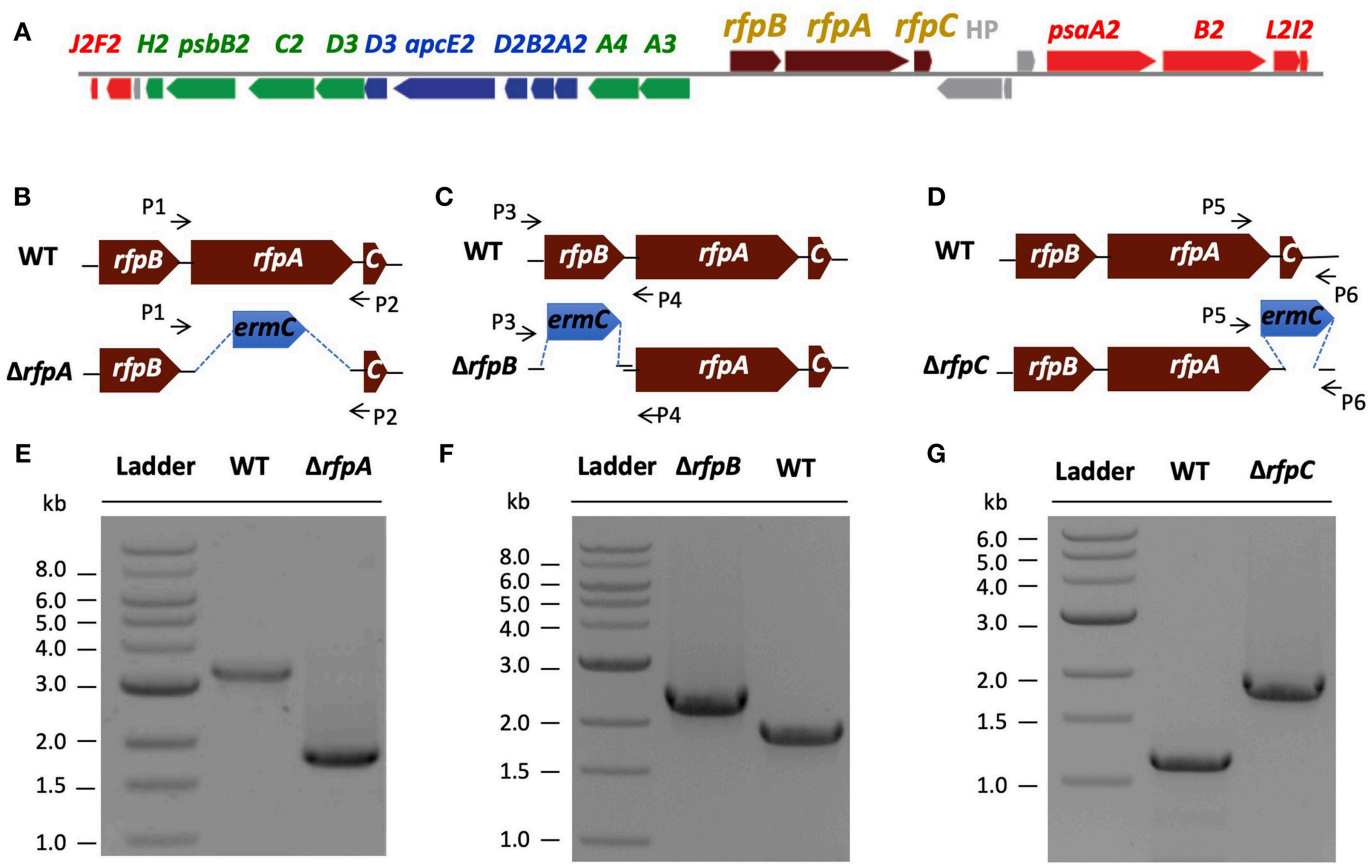
**The organization of the FaRLiP gene cluster in *Chl. fritschii* PCC 9212 and construction and validation of *rfpA*, *rfpB*, and *rfpC* deletion mutants**. **(A)** Gene organization of the FaRLiP gene cluster in *Chl. fritschii* PCC 9212. Red boxes represent genes encoding core subunits of PS I; green boxes represent genes encoding core subunits of PS II; blue boxes represent genes encoding core components of the phycobilisome; brown boxes represent regulatory *rfp* genes; and gray boxes represent genes that are not found in other FaRLiP clusters. **(B)** Schematic showing deletion of *rfpA*. The small arrows (P1 and P2) indicate the positions of the primers used for PCR verification of deletion. **(C)** Schematic showing deletion of *rfpB*. The small arrows (P3 and P4) indicate the positions of primers used for PCR verification of the deletion. **(D)** Schematic showing deletion of *rfpC*. The small arrows (P5 and P6) indicate the positions of primers used for PCR verification of the deletion. **(E)** Agarose gel electrophoresis of amplicons showing complete segregation of wild-type and mutant *rfpA* alleles. **(F)** Agarose gel electrophoresis of amplicons showing complete segregation of wild-type and mutant *rfpB* alleles. **(G)** Agarose gel electrophoresis of amplicons showing complete segregation of wild-type and mutant *rfpC* alleles.

**Figure 2:**
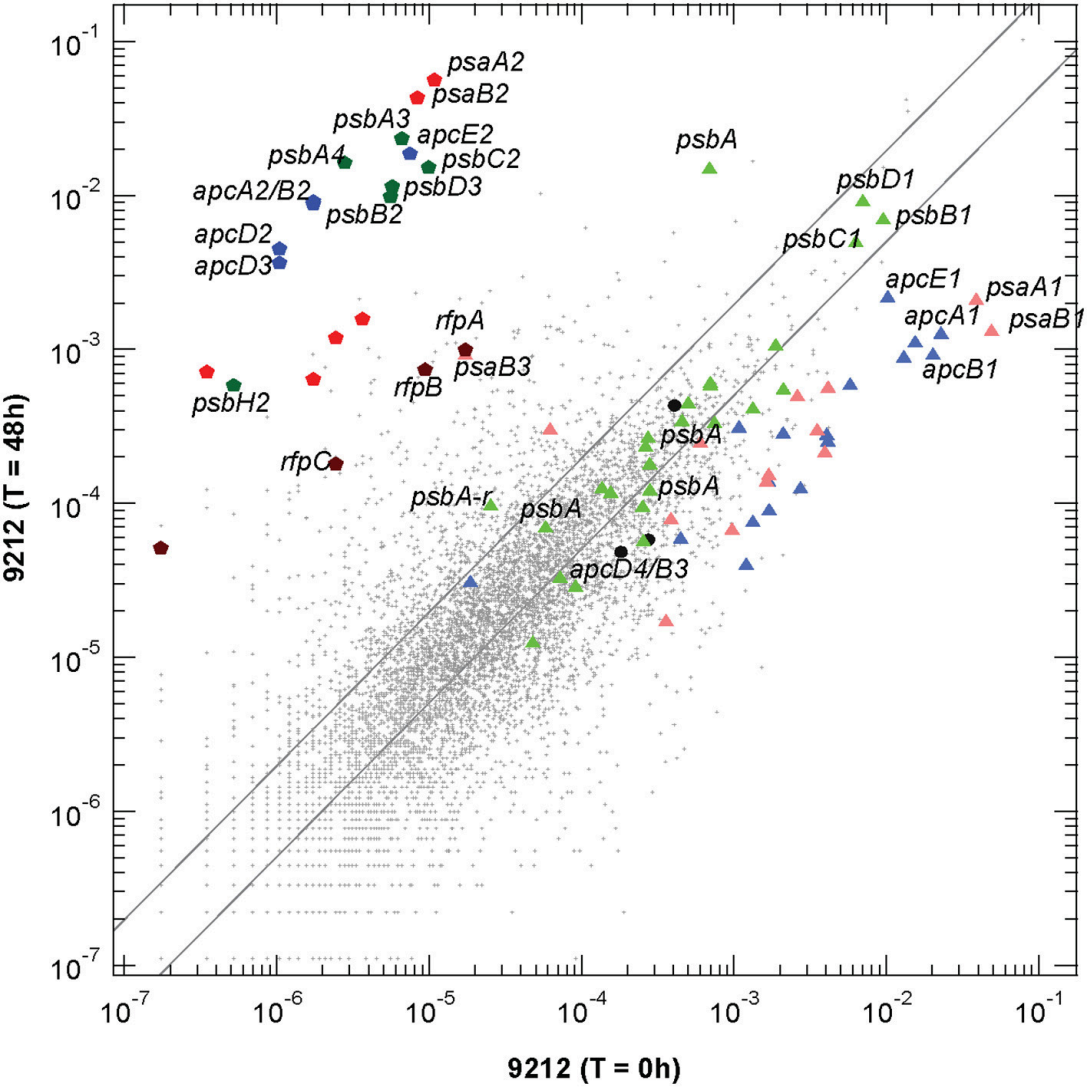
**Scatter plot of relative transcript abundances for *Chl*. *fritschii* PCC 9212 cells grown for 48 h in FRL compared to the *T* = 0 control (white light)**. The pentagon symbols represent the genes shown in Figure 1A with the same color coding. Triangular symbols represent genes expressed independent of light quality (i.e., white light). Green triangles represent genes encoding PS II subunits; pale red triangles represent genes encoding PS I subunits; and blue triangles represent genes encoding phycobiliproteins and related proteins (e.g., linker proteins). The three black circles represent the *apcD3-apcB4-isiX* gens of the LoLiP gene cluster (see text and Table S2 for more details). The parallel lines indicate a two-fold increase and a 50% decrease in relative transcript abundance.

In a previous study, we established that *Chl. fritschii* PCC 9212 can grow in FRL and can synthesize Chl *d*, Chl *f*, and FRL-absorbing phycobiliproteins (Gan et al., 2015). This cyanobacterium contains a complete FaRLiP gene cluster that includes the 17 common FaRLiP genes encoding core components of the photosynthetic apparatus (Figure 1A) as well as the *rfpBAC* operon. To obtain a more comprehensive view of the FaRLiP response in *Chl. fritschii* PCC 9212, we performed a transcription profiling study of this strain. Table S2 presents the complete data for samples collected from cells grown in WL as well as from cells grown in FRL for 12, 24, 48, and 336 h. Figure 2 shows that photosynthesis genes can be described as belonging to one of two families: the FaRLiP paralogs (upper left quadrant, darker colors) which are *only* expressed in FRL, and the “WL paralogs,” which are transcribed in both WL and FRL but are responsible for the photosynthetic apparatus found in cells grown in WL (lighter colored symbols). Some “WL paralogs” are single-copy genes and thus must be transcribed under both conditions to produce active photosynthetic complexes. The true “WL paralogs” that have homologs in the FaRLiP cluster generally show reduced transcription in FRL, but their transcription still occurs (this will be important in interpreting RT-PCR data in **Figure 6** below). The enormous difference in transcript levels of the FaRLiP paralogs relative to the “WL paralogs” accounts for the remodeling of the photosynthetic apparatus that occurs in cells grown in FRL.

Transcripts for the FaRLiP gene cluster are already elevated by 12 h and reached a maximum at about 48 h. Figure 2 shows a scatter-plot comparison of the relative transcript abundances for *Chl. fritschii* PCC 9212 cells grown in FRL for 48 h compared to cells grown in WL. The transcript levels for many genes unrelated to photosynthesis increased by more than two-fold (~972 total genes), and transcript abundances for many genes decreased by more than 50% (~2720 total genes; see Table S2 for details). Thus, more than half (~55%) of genes in this organism change transcript level by at least a factor of two when cells are grown in FRL for 48 h. Thus, the FaRLiP response represents a major restructuring of not only the photosynthetic apparatus but of cellular composition and metabolism in general, as reported for *Leptolyngbya* sp. JSC-1 (Gan et al., 2014).

The dark-colored pentagonal markers in Figure 2 indicate the photosynthesis genes encoded in the FaRLiP gene cluster, with blue pentagons representing core subunit genes of the phycobilisome, green pentagons representing core subunit genes of PS II, and red pentagons representing core subunit genes of PS I. The dark brown pentagons represent the *rfp* genes (note that this organism has genes encoding two CheY-like receptors in the gene cluster shown in Figure 1A: *rfpC*, and a second gene that shows ~62% sequence similarity with *rfpC*, so four genes are represented on Figure 2. Among these genes, the two most highly transcribed genes, *psaA2* and *psaB2*, show an increase of >5200-fold, and even the regulatory *rfp* genes show increases of more than 50-fold (see Table S2). This latter observation strongly implies that the *rfp* genes are auto-regulated and that cells produce more of these regulatory proteins when they are grown in FRL. The triangular symbols, using similar coloring for the photosynthetic complexes, represent the paralogous genes for subunits of complexes that are normally expressed in WL (or that are expressed in a light-independent manner; most of these genes are also expressed in FRL). The majority of the genes for PS I and PBP subunits have substantially lower relative transcript abundances in cells grown in FRL. For example, the *psaA1* and *psaB1* transcripts are about 37-fold and 19-fold less abundant (Table S2). On the other hand, transcript levels for many PS II genes are similar under WL and FRL. The three black circles represent the *apcD4-apcB3-isiX* genes of the LoLiP gene cluster (Olsen et al., 2015). Note that relative transcript levels for these three genes did not increase in cells grown in FRL.

### Construction and validation of *rfpA, rfpB*, and *rfpC* mutants

A major, unresolved issue from initial studies of FaRLiP was whether the products of the *rfpA-rfpB-rfpC* genes are the master control elements for this acclimation response. To address this question, we used biparental conjugation with *E. coli* to introduce DNA into *Chl. fritschii* PCC 9212 and to delete the *rfpA, rfpB*, and *rfpC* genes (see Figures 1B–D). An *ermC* gene cassette (Em^R^) was used to replace each gene individually. After mating, selection, and repeated streaking, antibiotic-resistant transconjugants were obtained. Figure 1E shows the results of analytical agarose gel electrophoresis of PCR amplicons derived using forward and reverse primers for *rfpA* (see Table S1) with WT and transconjugant DNAs as templates. The results show that complete gene replacement had occurred without sucrose counter-selection and that wild-type and mutant alleles had fully segregated. Thus, RfpA is not essential for cell viability when cells are grown in WL. Figures 1F,G show similar PCR results for the *rfpB* and *rfpC* genes, which likewise are not essential for cell viability when cells are maintained in WL.

### Physiological characterization of Rfp mutants

**Figure 3:**
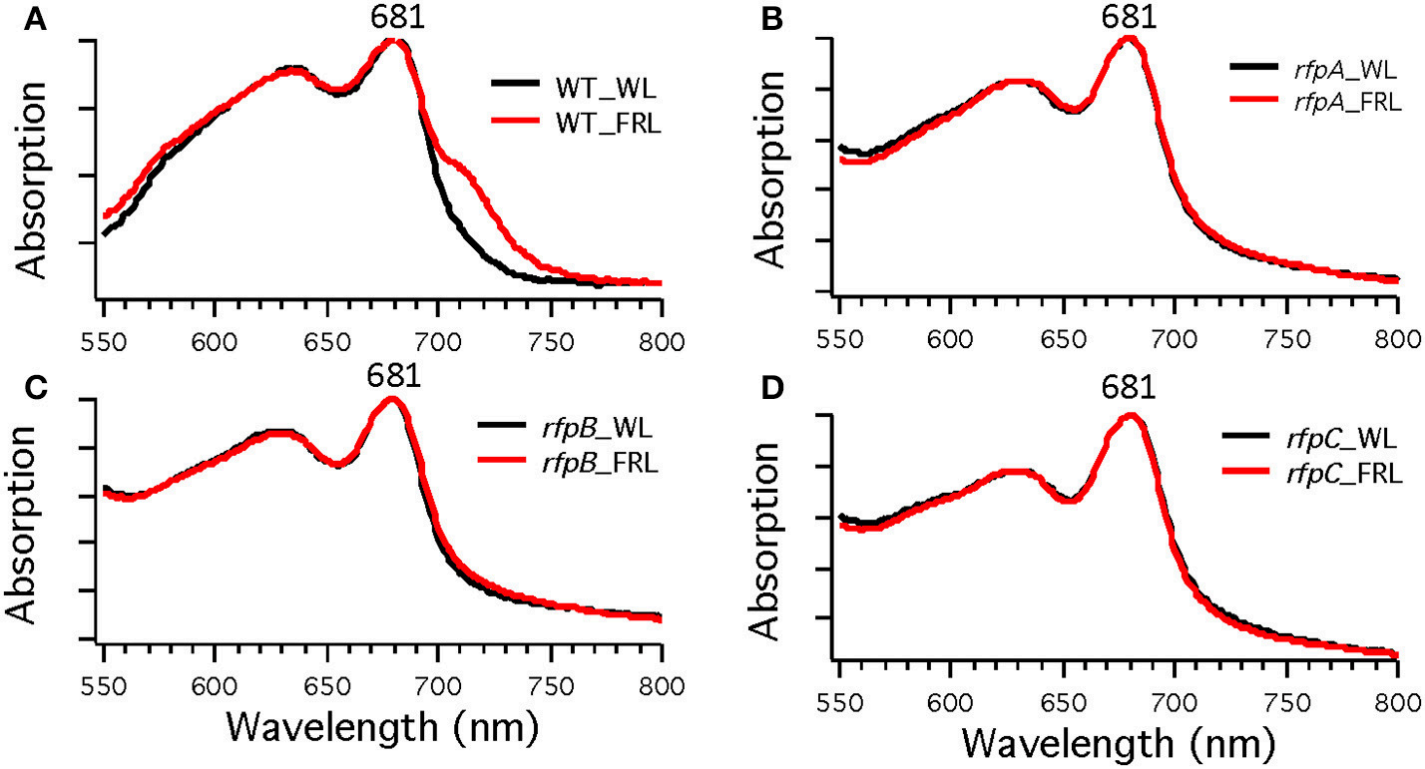
**Whole-cell absorption spectra of *Chl. fritschii* PCC 9212 wild-type (WT) in (A), *rfpA* mutant in (B), *rfpB* mutant in (C), and *rfpC* in (D)**. Cells were grown in white light (black lines) or grown in FRL (red lines).

**Figure 4:**
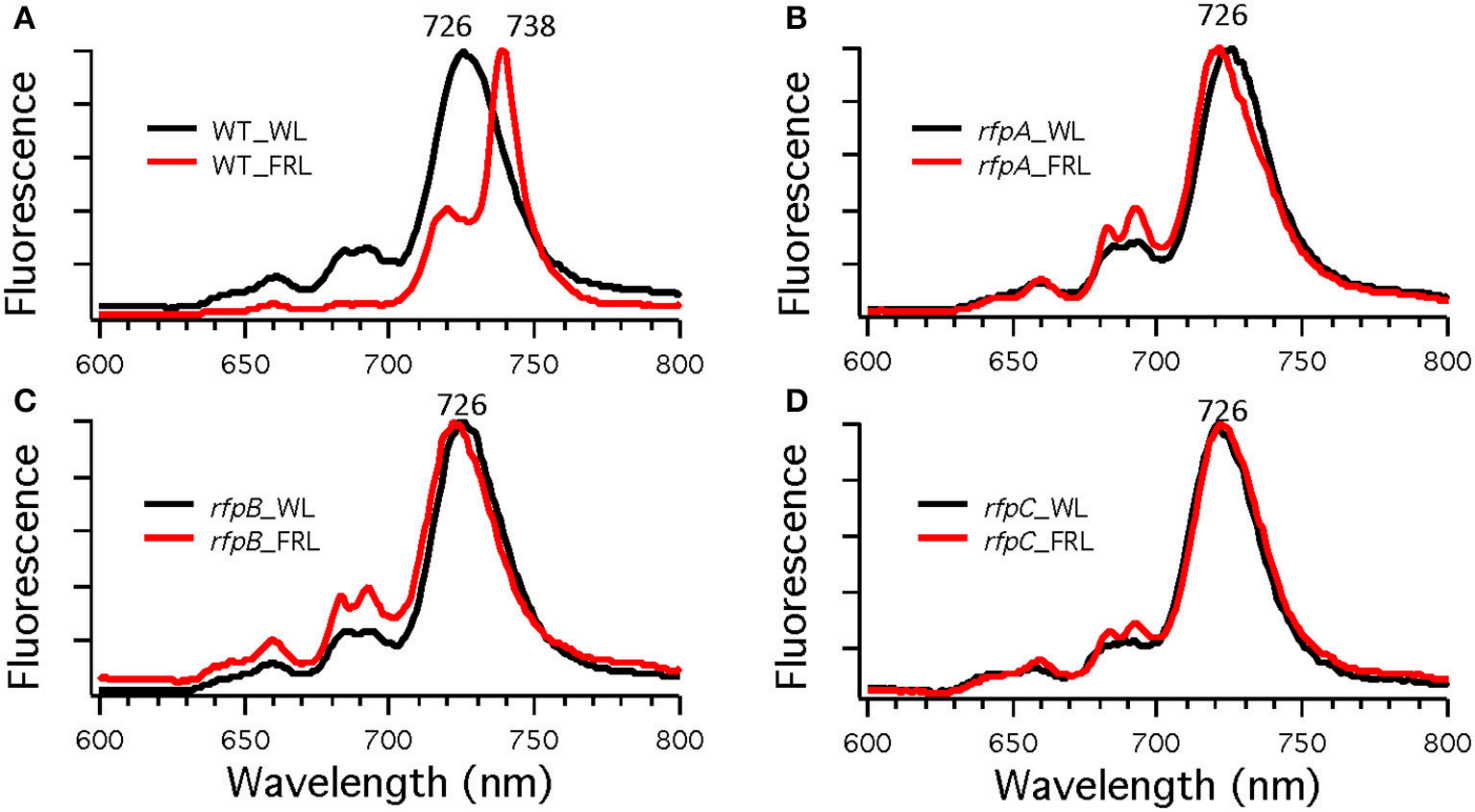
**Low-temperature (77 K) fluorescence emission spectra of of *Chl. fritschii* PCC 9212 wild-type (WT) in (A), *rfpA* mutant in (B), *rfpB* mutant in (C), and *rfpC* in (D)**. Cells were grown in white light (WL; black lines) or grown in far-red light (FRL; red lines). The excitation wavelength was 440 nm to excite Chls preferentially.

To assess the effects of deleting the *rfpA, rfpB*, and *rfpC* genes, wild type and mutant cells were grown in WL and then transferred to FRL. After 4 days of growth, whole-cell absorption and low-temperature fluorescence emission spectra were recorded. As shown in Figure 3, only the wild-type strain could alter its photosynthetic apparatus to increase absorption above 700 nm when cells were grown in FRL. The wild-type cells grown in FRL also showed a fluorescence emission band with a maximum at 739 nm (Figure 4A), which is indicative of the synthesis of Chl *f* and the biogenesis of new photosystem complexes (Gan et al., 2015). In contrast, fluorescence emission spectra for the three mutant strains (Figures 4B–D) showed no evidence that Chl *f* was being synthesized when cells were incubated in FRL. Increasing the time of incubation in FRL did not lead to Chl *f* synthesis or to changes in the photosynthetic apparatus for the mutant strains. In fact, the mutant cultures slowly became chlorotic over time because the cells were unable to grow in FRL.

**Figure 5:**
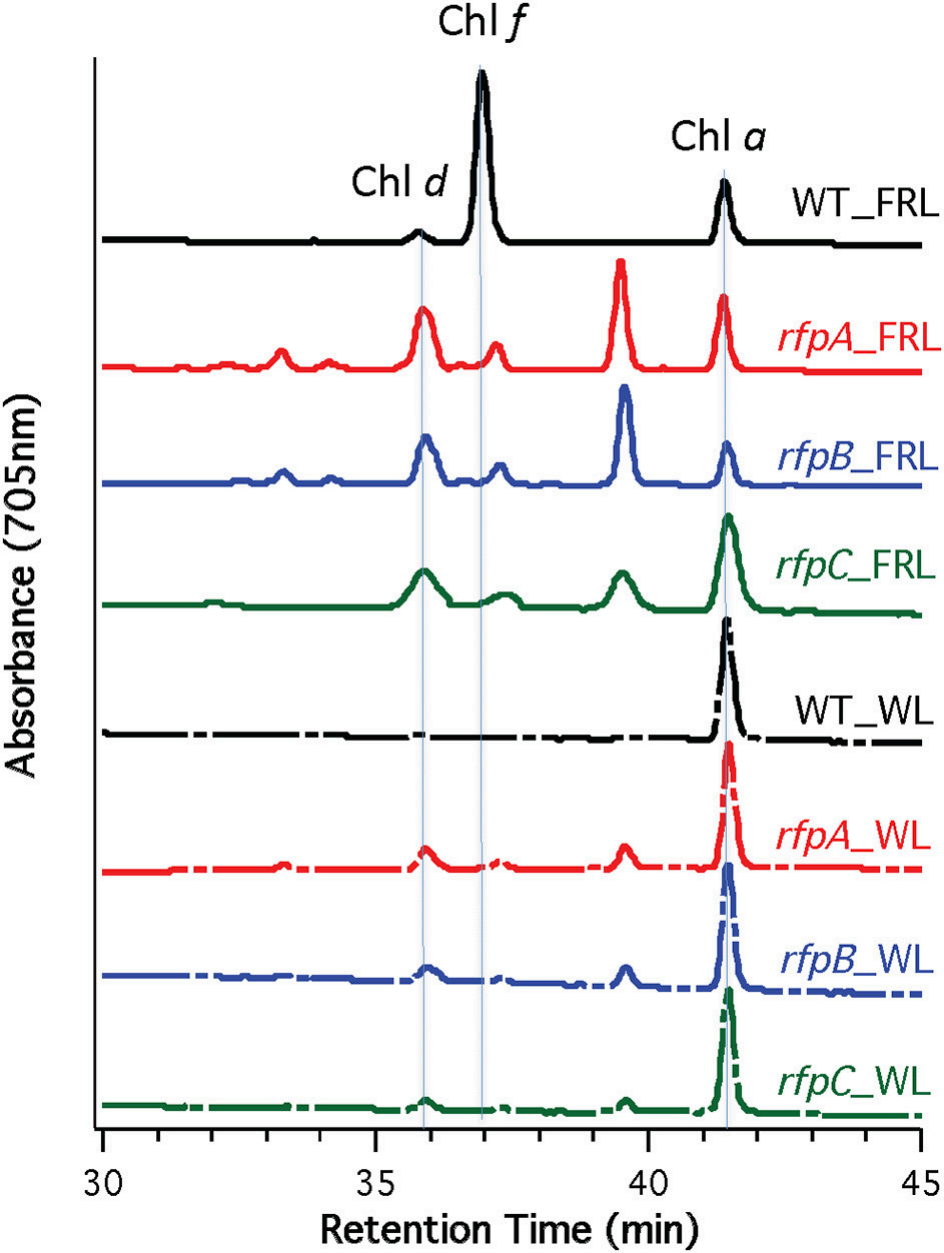
**HPLC elution profile monitored at 705 nm of pigment from the wild type (WT) and *rfp* mutant strains of *Chl. fritschii* PCC 9212 grown in white light (WL) and far-red light (FRL)**. The elution positions of Chl *d*, Chl *f*, and Chl *a* are indicated. The unidentified peaks had the absorption spectrum of Chl *a*, but were less hydrophobic than Chl *a*. These are probably Chl *a* esterified with alcohols less saturated than phytol.

Figure 5 shows HPLC elution profiles monitored at 705 nm, which is the peak absorption value for Chl *f* in the solvents used for the analysis. As expected, wild-type cells grown in WL only synthesized Chl *a*, but wild-type cells grown in FRL showed peaks characteristic of Chl *d*, Chl *f*, and Chl *a* at 36, 37, and 42 min, respectively. Analyses of Chls in cells of the *rfpA, rfpB* and *rfpC* mutant grown in WL surprisingly showed the presence of both Chl *d* and Chl *a*, but importantly, no Chl *f* was detected in cells grown in WL or FRL. However, when the mutants were grown in FRL, two new peaks were observed, eluting at 37.5 and 39 min, both of which had absorption spectra identical to that of Chl *a*. From their elution behavior, these pigments are more hydrophilic than Chl *a* and mostly likely represent Chl *a* esterified with alcohols more unsaturated than phytol. These alternative forms of Chl *a* were also observed when wild-type cells were grown photoheterotrophically, and they have also been observed in *Synechococcus* sp. PCC 7335 (data not shown). The significance of these forms of Chl *a* is presently unknown.

### RT-PCR analyses of FaRLiP gene expression

**Figure 6:**
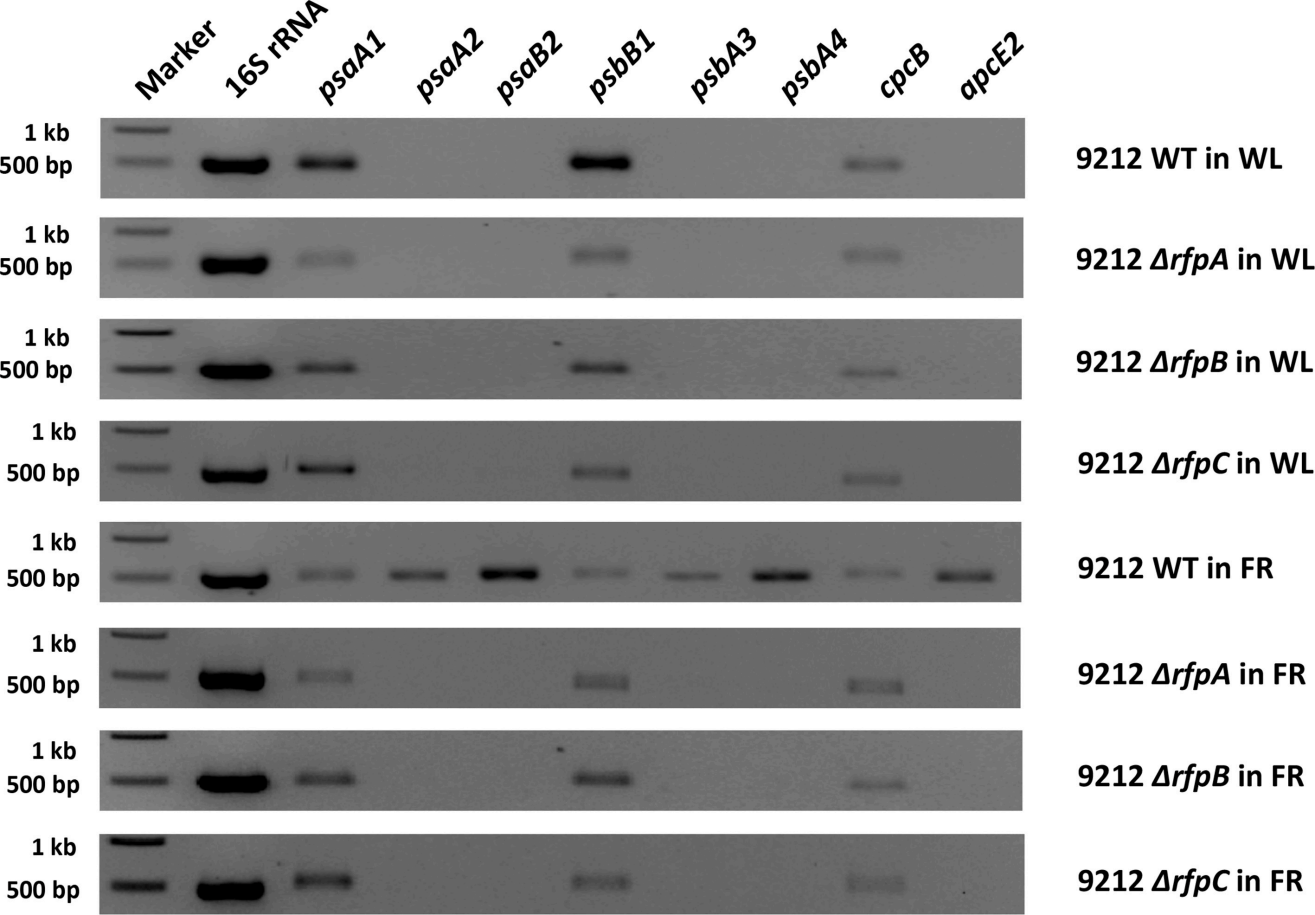
**Agarose gel electrophoresis of amplicons produced by RT-PCR using template RNAs from the indicated strains of *Chl. fritschii* PCC 9212 grown in white light (WL) or far-red light (FRL)**. Genes *psaA2, psaB2, psbA4, psbA5*, and *apcE2* are found in the FaRLiP gene cluster (see Figure 1A) and are specifically expressed in FRL in the wild type but not in any of the mutant strains. Genes *psaA1, psbB1*, and *cpcB* are expressed under all conditions in all strains because they are not part of the FaRLiP response.

To assess whether RfpA, RfpB, and RfpC are involved in controlling transcription of the FaRLiP gene cluster, we isolated RNA from wild-type and mutant cells grown in WL or FRL and performed reverse-transcriptase- (RT) PCR assays. Five genes from the FaRLiP gene cluster were selected for analysis: *psaA2, psaB2, psbA3, psbA4*, and *apcE2*. As controls, three paralogous genes normally expressed in WL were also analyzed: *psaA1, psbB1*, and *apcE1*. As expected, transcripts were not detected for genes from the FaRLiP cluster when wild-type cells were grown in WL, but transcripts for all five genes were highly abundant in RNA isolated from wild-type cells grown in FRL (Figure 6). By varying the number of cycles in the PCR reactions, we estimated that these transcripts were at least 1000-fold more abundant under these conditions, which agrees well with the data in Figure 2. In contrast, no transcripts were detected in RNA isolated from the *rfpA, rfpB*, or *rfpC* mutant strains when cells were grown in FRL or WL. Combining previous results on RfpA showing it is a red/far-red phytochrome (Gan et al., 2014), and domain analysis of RfpB (see Introduction), these results confirm that RfpB is a transcription activator for the genes of the FaRLiP gene cluster, and that RfpA is the light sensor for this acclimation process. In contrast, transcripts for the paralogous genes normally expressed in WL were detected in RNA samples for the wild type and the mutants regardless of the growth light conditions. These results are also consistent with the transcription profiling results shown in Figure 2, and they establish that the RNA samples were not degraded. Other control experiments omitting reverse transcriptase showed that the RNA templates were free from contaminating DNA (data not shown). Collectively, these results establish that RfpA, RfpB, and RfpC are the master control elements of the FaRLiP response.

**Figure 7:**
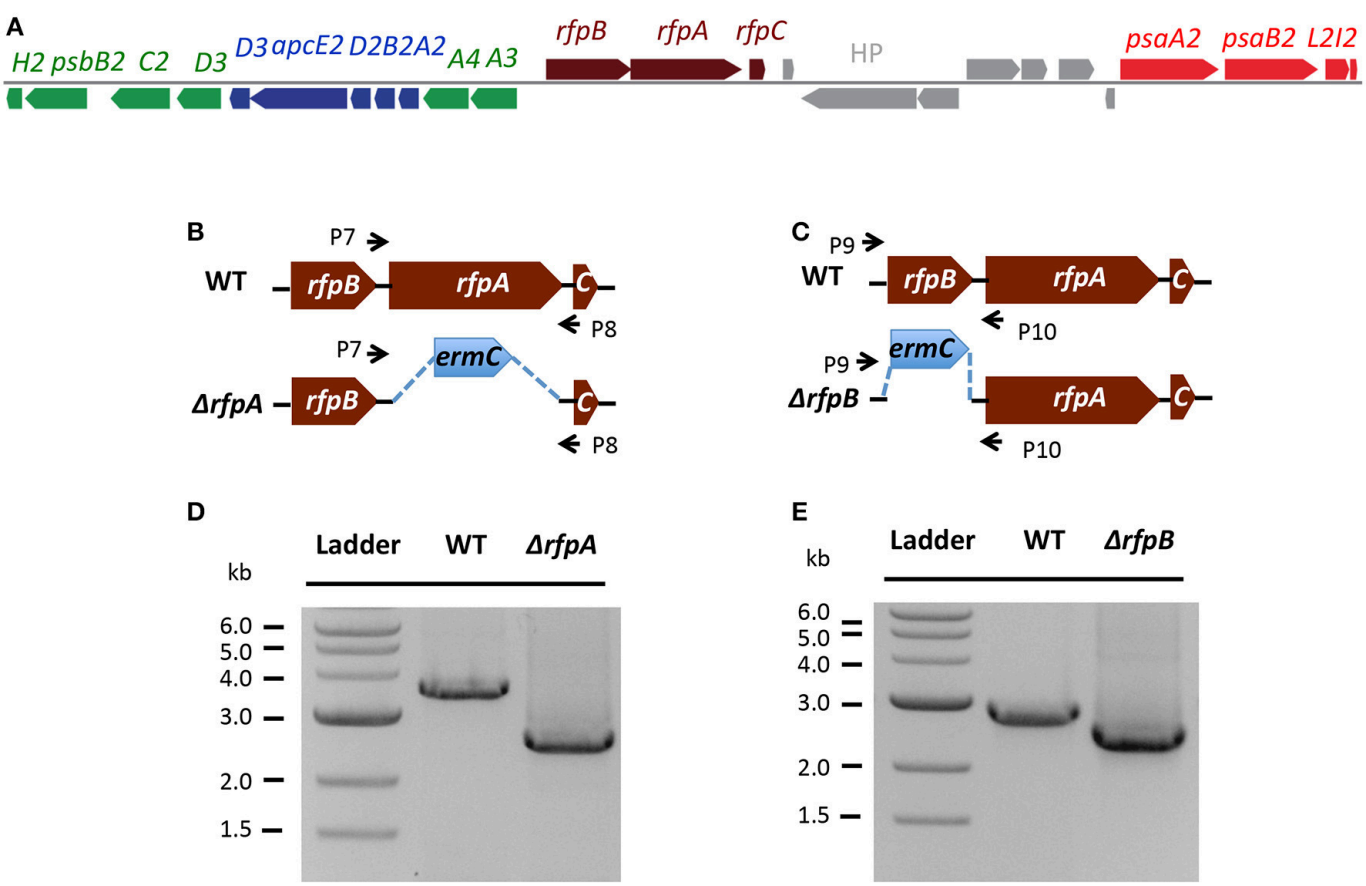
**The organization of the FaRLiP gene cluster in *Chr. thermalis* PCC 7203 and construction and validation of *rfpA* and *rfpB* deletion mutants**. **(A)** Organization of the FaRLiP gene cluster. Red boxes represent genes encoding core subunits of PS I; green boxes represent genes encoding core subunits of PS II; blue boxes represent genes encoding core components of the phycobilisome; brown boxes represent regulatory *rfp* genes; and gray boxes represent hypothetical genes. **(B)** Schematic showing deletion of *rfpA*. The small arrows (P7 and P8) indicate the positions of the primers used for PCR verification of deletion. **(C)** Schematic showing deletion of *rfpB*. The small arrows (P9 and P10) indicate the positions of primers used for PCR verification of the deletion. **(D)** Agarose gel electrophoresis of amplicons showing complete segregation of wild-type and mutant *rfpA* alleles. **(E)** Agarose gel electrophoresis of amplicons showing complete segregation of wild-type and mutant *rfpB* alleles.

**Figure 8:**
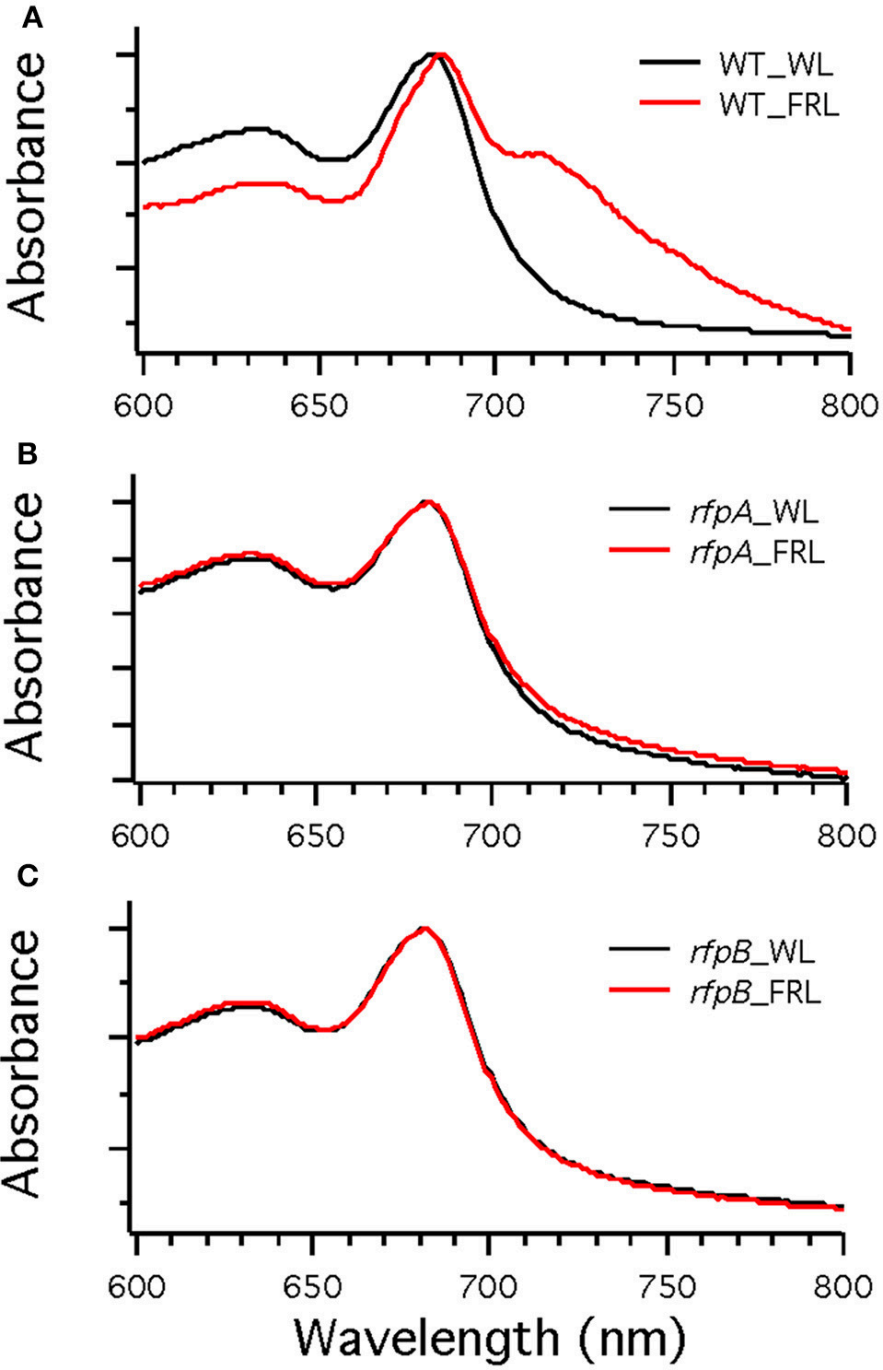
**Whole cell absorption spectra of wild-type (WT) (A) and the *rfpA* (B) and *rfpB* (C) mutant strains of *Chr. thermalis* PCC 7203**. Cells were grown in white light (WL; black lines) or far-red light (FRL; red lines). Note that only the WT can modify its photosynthetic apparatus when grown in FRL.

**Figure 9:**
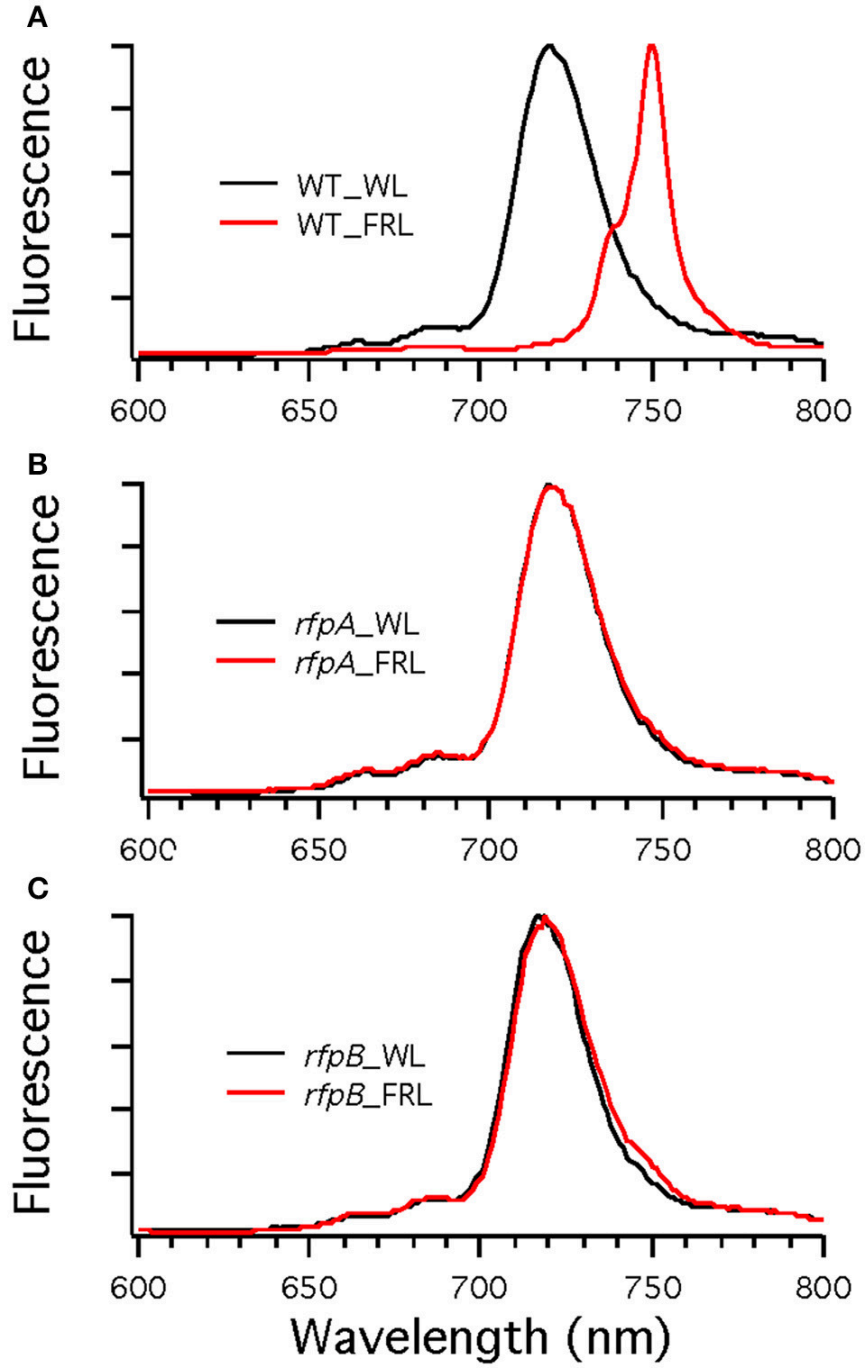
**Low-temperature (77 K) fluorescence emission spectra of wild type (WT) (A) and *rfpA* (B) and *rfpB* (C) mutant strains of *Chr. thermalis* PCC 7203 grown in white light (WL; black lines) or far-red light (FRL; red lines)**. The excitation wavelength was 440 nm to excite Chls preferentially. Note that only the WT cells grown in FRL have long-wavelength emission characteristic of Chl *f*.

**Figure 10:**
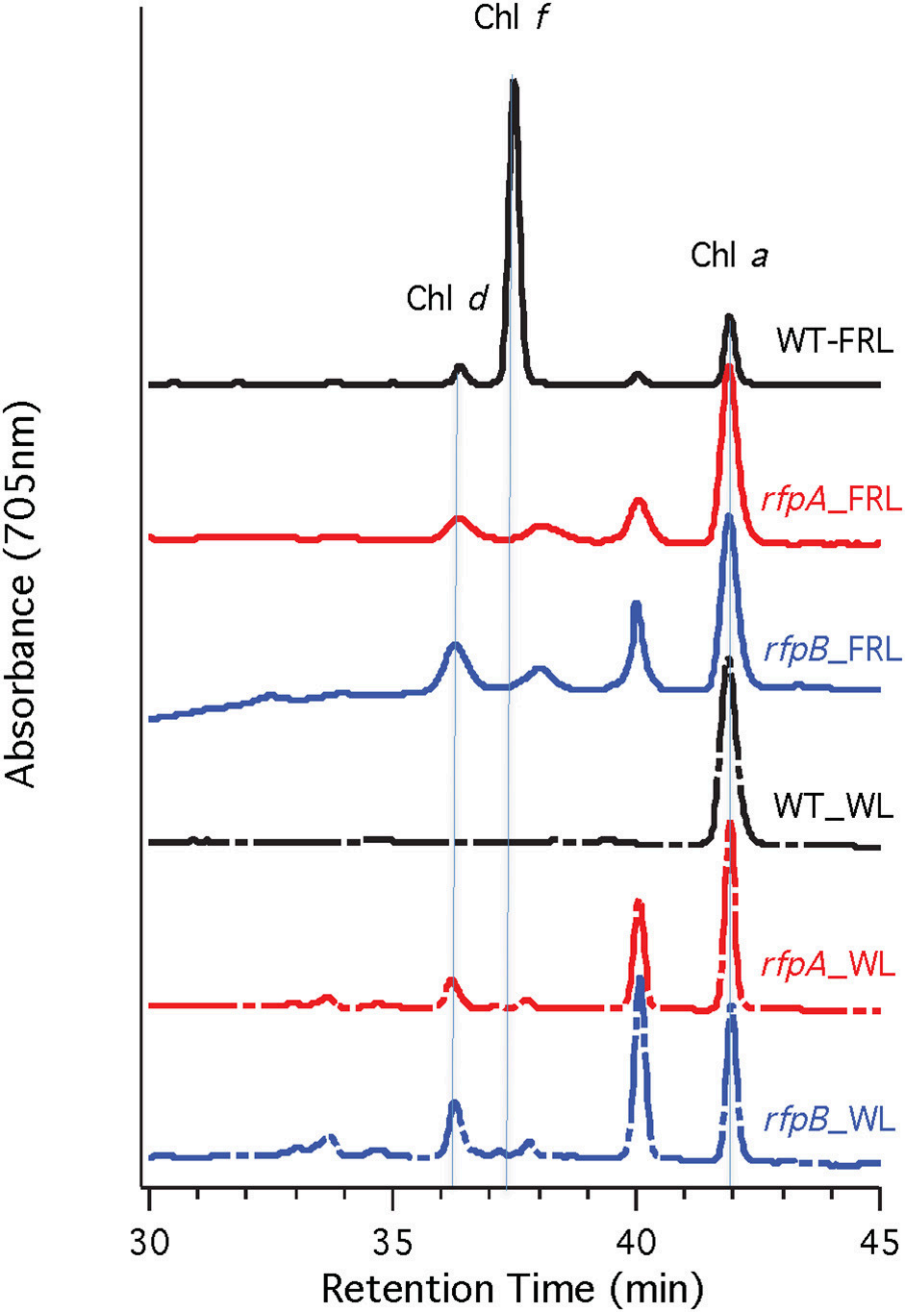
**HPLC elution profile monitored at 705 nm of pigments from the wild type (WT) and *rfpA* and *rfpB* mutant strains of *Chr. thermalis* PCC 7203 grown in white light (WL) and far-red light (FRL)**. The elution positions of Chl *d*, Chl *f*, and Chl *a* are indicated by the vertical dotted lines. The unlabeled peaks at 37.5 and 40 min had the absorption spectrum of Chl *a* and likely represent Chl *a* esterified with alcohols less saturated than phytol.

Figure 7A shows the arrangement of genes in the FaRLiP cluster of *Chr. thermalis* PCC 7203. Overall, this cluster is very similar to that in *Chl. fritschii* PCC 9212, but two PS I genes, *psaJ2* and *psaF2*, are missing from the cluster and are located elsewhere on the genome. Previous studies showed that conjugation from *E. coli* could be used to introduce DNA into some *Chroococcidiopsis* spp., although *Chr. thermalis* PCC 7203 was not specifically tested (Billi et al., 2001; Billi, 2010). Deletion of the *rfpA* and *rfpB* genes from *Chr. thermalis* PCC 7203 confirmed that RfpA and RfpB are the master control elements for FaRLiP in this organism as well. Briefly, fully segregated mutants were obtained for both genes (see Figures 7B–E). When these mutants were grown in FRL, cells were unable to increase their absorption above 700 nm (Figure 8), did not exhibit fluorescence emission characteristic of Chl *f* (Figure 9), were unable to synthesize Chl *f* (Figure 10) and as a result were unable to grow in FRL. However, as observed for *Chl. fritschii* PCC 9212, inactivation of the *rfpA* and *rfpB* genes in *Chr. thermalis* PCC 7203 led to derepression of Chl *d* synthesis in WL (Figure 10). These results are in complete agreement with those obtained for *Chl. fritschii* PCC 9212 and suggest that regulation of the FaRLiP response in other FaRLiP cyanobacteria will be similar to that described here.

## Discussion

The FaRLiP acclimation response is a transcription program triggered by FRL that leads to an extensive remodeling of the photosynthetic apparatus of cells that have the ability to synthesize Chl *f* and Chl *d* and that have the requisite gene cluster. The synthesis of paralogous forms of PS I, PS II, and phycobiliprotein core proteins, in addition to the synthesis of Chl *f* and Chl *d*, produces cells with enhanced absorption between 700 and 800 nm, and these changes collectively enables cells to grow in FRL (Gan et al., 2014, 2015; Gan and Bryant, 2015). Based upon the results obtained in this study, RfpA, RfpB, and RfpC are critically important regulatory proteins in the FaRLiP response.

**Figure 11:**
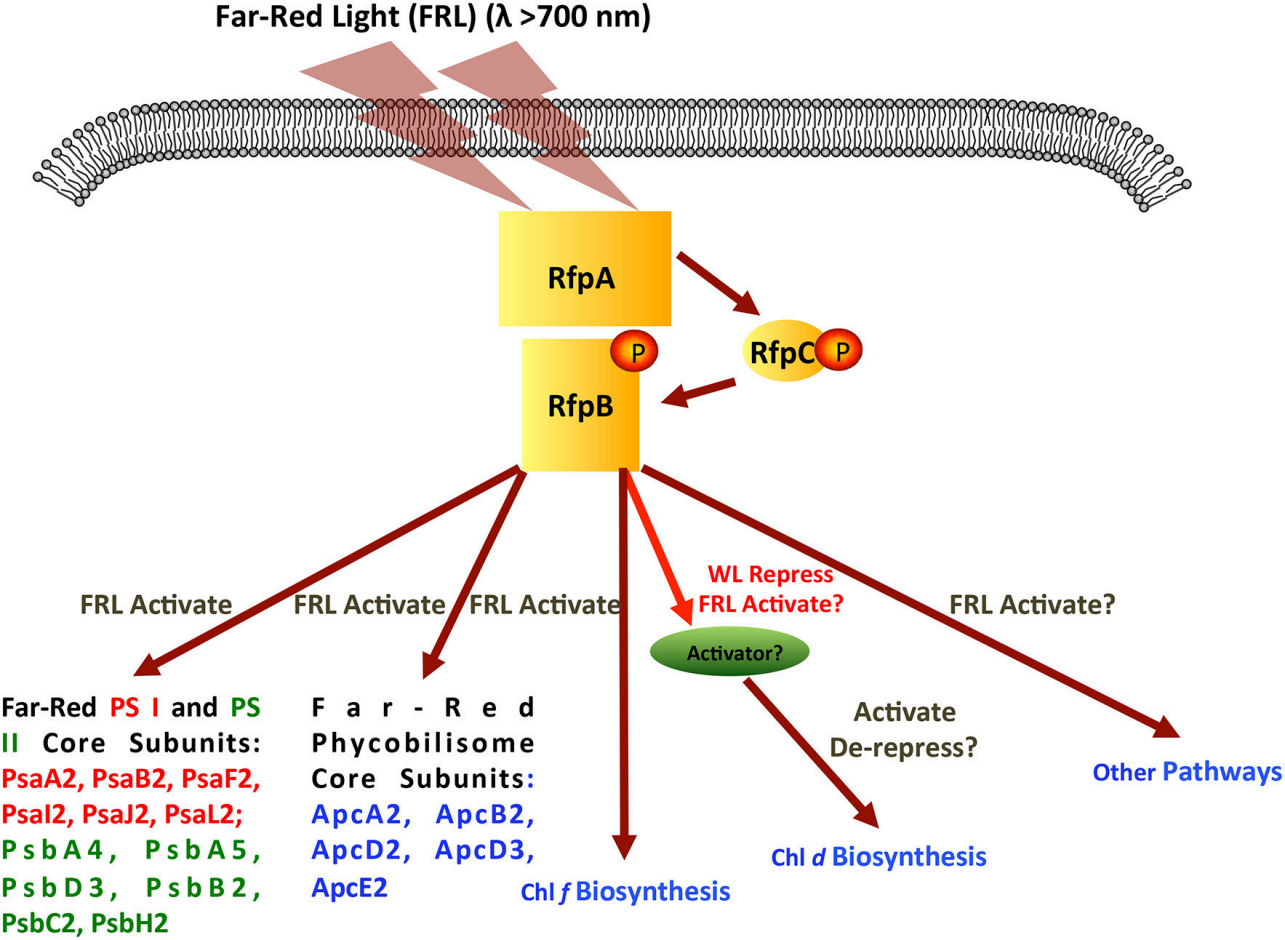
**Scheme showing the signal transduction cascade formed by RfpA, RfpB, and RfpC**. Although the figure shows the RfpB being activated by phosphorylation, it is possible that the active form would be dephosphorylated. In this model, far-red light activates the histidine kinase of RfpA, which in turn phosphorylates RfpC, and finally phosphorylates the response regulator/transcription activator RfpB. Genes and process regulated by activated RfpB are indicated.

Figure 11 shows a model for regulation of FaRLiP response by RfpA, RfpB, and RfpC. RfpA is a knotless phytochrome, which forms a unique structural class within the large family of cyanobacterial photoreceptors (Gan et al., 2014). RfpA has the domain structure GAF-PHY-PAS-Histidine-Kinase, which is distinct from that of the knotted phytochrome photoreceptor, RcaE, of complementary chromatic acclimation, which has the domain organization PAS-GAF-PAS-Histidine-Kinase (Gutu and Kehoe, 2012; Gan et al., 2014). Thus, these two photoreceptors that control photoacclimation responses in cyanobacteria are not closely related evolutionarily. It appears that these two acclimation responses arose independently, and there is no overlap in the genes controlled by the two photoreceptors. To date, orthologs of RfpA are only found in the genomes of the 13 cyanobacteria that possess FaRLiP gene clusters and RcaE has only been observed in some but not all cells that synthesize phycoerythrin. We previously showed that the GAF domain of RfpA binds a phycocyanobilin chromophore to form a red/far-red responsive photoreceptor (Gan et al., 2014). A broad range of light wavelengths can produce the far-red-absorbing (P_fr_) form of RfpA, but only FRL (λ≥700 nm) produces the red absorbing (P_r_) form (see Gan et al., 2014). Thus, in the model shown in Figure 11, absorption of FRL activates the histidine kinase to transfer a phosphate to RfpC, which in turn transfers the phosphate to RfpB. RfpC is a member of the CheY family and is likely to undergo reversible phosphorylation. Note that FRL could also inactivate the histidine kinase, which would allow a phosphatase activity to remove the phosphate from RfpB and RfpC. At present, we do not know which state, phosphorylated or unphosphorylated, of RfpB is the transcriptional activator, nor do we know the exact role of RfpC, but these details can be ascertained from future studies.

RfpB is a DNA-binding response regulator, which contains two CheY receiver domains that flank a winged-helix, DNA-binding domain. RfpB apparently acts as a transcription activator of all of the genes of the FaRLiP cluster, because deletion of the *rfpB* gene did not lead to derepression of any of the genes of the FaRLiP gene cluster. RfpB either directly or indirectly must also regulate the genes for the synthesis of Chl *f*, which was not synthesized by the mutants in WL or FRL. Surprisingly, the synthesis of Chl *d* was de-repressed in all *rfp* mutants in both cyanobacteria tested. This resulted in the inappropriate synthesis of Chl *d* in cells grown in WL.

The results presented here establish that conjugation can be used to delete genes in *Chl. fritschii* PCC 9212 and in *Chr. thermalis* PCC 7203. These studies confirm and significantly extend the results of Stucken et al. (2012) and Billi et al. (2001), who demonstrated conjugation-based transfer of DNA into *Chl. fritschii* PCC 6912 and *Chroococcidiopsis* spp., respectively. In other studies, we have found that conjugation also works well in *Synechococcus* sp. PCC 7335 (M.-Y. Ho, C. Zhao, and D. A. Bryant, unpublished results), and we have produced *rfpA, rfpB*, and *rfpC* mutants in this strain as well. We are currently testing whether conjugation can be used to produce mutants in *Fischerella thermalis* PCC 7521, a thermophilic FaRLiP strain that could be used to produce protein complexes for crystallization. *Fischerella* (*Mastigocladus*) spp. have previously been used successfully for this purpose (Almog et al., 1991; Kurisu et al., 2003; Kim et al., 2013). Thus, it should be possible to use mutational and biochemical analyses to characterize the modified photosynthetic complexes produced in diverse FaRLiP strains grown in FRL.

Although several options are now available for strains that could serve as model organisms for the FaRLiP response, *Chl. fritschii* PCC 9212 appears to be the optimal choice. *Chlorogloeopsis* spp. are filamentous but this aspect is often only apparent in hormogonia, which form short chains of barrel-shaped cells (Evans et al., 1976). After losing motility, these cells enlarge to become spherical vegetative cells within an extracellular sheath. Heterocysts develop both in terminal and intercalary positions. As growth continues, cell division in more than one plane occurs, and multiseriate trichomes form, which causes loss of the filamentous nature as the trichomes fragment into amorphous aggregates. *Chlorogloeopsis* spp. are very good chemoheterotrophs, growing well on sucrose as well as acetate and other sugars, including glucose, fructose, and ribose (Hoffman and Castenholz, 2001; Zhang and Bryant, 2015). Interestingly, growth on sucrose suppresses the formation of trichomes (Evans et al., 1976), a trait that could improve the efficiency of conjugation. *Chl. fritschii* PCC 9212 has gene clusters for both the FaRLiP and LoLiP responses, and it grows well on plates and in liquid media. These properties make it an excellent choice as a model organism for studies of FaRLiP and LoLiP.

## Author contributions

CZ, FG, and GS performed the research. DB directed the research and wrote the initial draft of the manuscript. All authors participated in editing and correcting the draft.

### Conflict of interest statement

The authors declare that the research was conducted in the absence of any commercial or financial relationships that could be construed as a potential conflict of interest.

## Abbreviations

Chl: chlorophyll
FaRLiP: Far-Red Light Photoacclimation
FRL: far-red light
HEPES: 4-(2-hydroxyethyl)-1-piperazineethanesulfonic acid
LoLiP: Low-Light Photoacclimation
WL: white light.
