# Corrigendum: RfpA, RfpB, and RfpC are the Master Control Elements of Far-Red Light Photoacclimation (FaRLiP)

**DOI:** 10.3389/fmicb.2019.00549

**Published:** 2019-03-22

**Authors:** Chi Zhao, Fei Gan, Gaozhong Shen, Donald A. Bryant

**Affiliations:** ^1^Department of Biochemistry and Molecular Biology, The Pennsylvania State University, University Park, PA, United States; ^2^Department of Chemistry and Biochemistry, Montana State University, Bozeman, MT, United States

**Keywords:** photosynthesis, photosystem I, photosystem II, phycobilisome, chlorophyll *f*, chlorophyll *d*, *Chlorogloeopsis fritschii* PCC 9212, *Chroococcidiopsis thermalis* PCC 7203

In the original article, there was a mistake in Figure 1 as published. In Panel F, the lane labeled “Δ*rfpB*” should have been labeled “WT”, while the lane labeled “WT” should have been labeled “Δ*rfpB”*. The corrected Figure 1 appears below.

**Figure 1:**
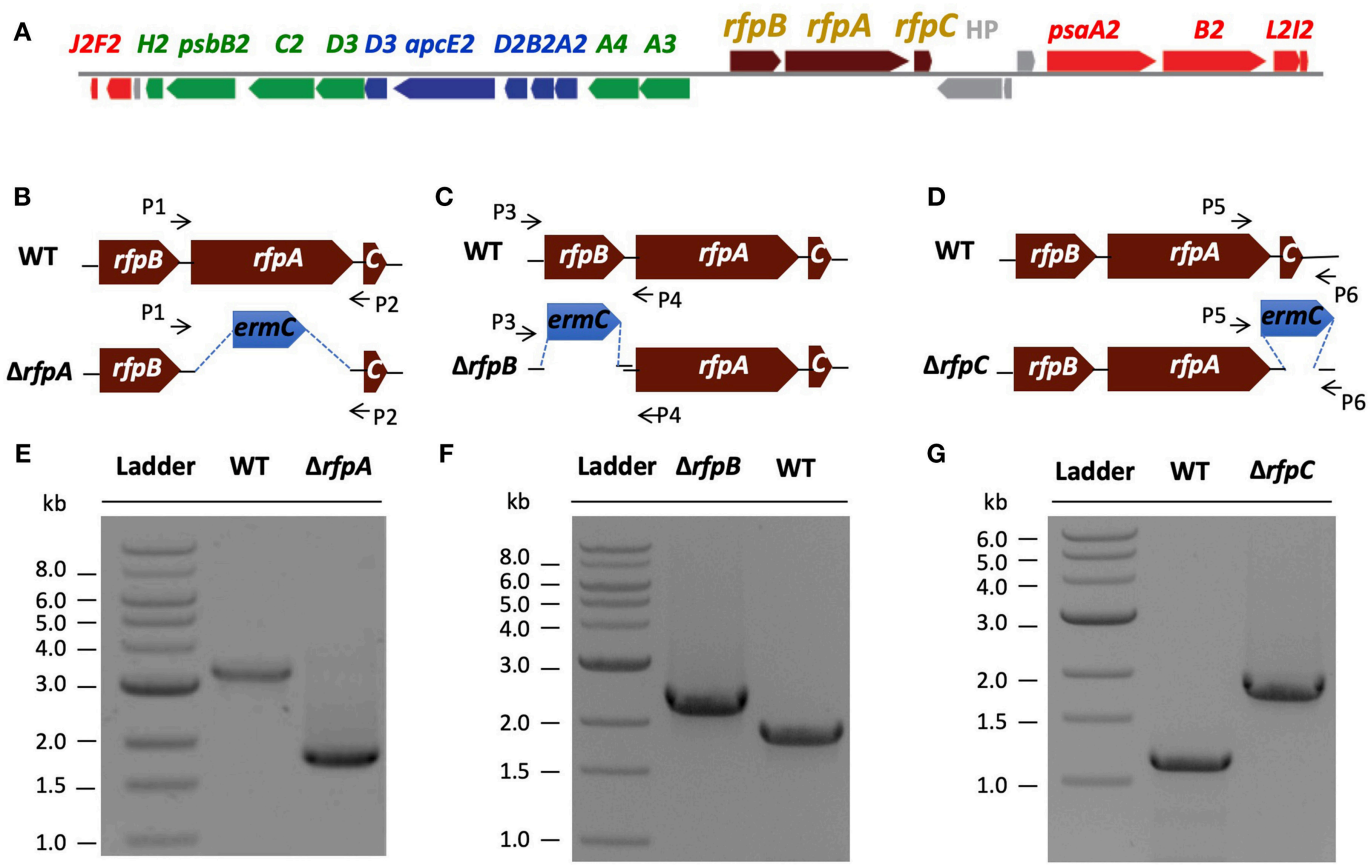
The organization of the FaRLiP gene cluster in *Chl. fritschii* PCC 9212 and construction and validation of *rfpA, rfpB*, and *rfpC* deletion mutants. **(A)** Gene organization of the FaRLiP gene cluster in *Chl. fritschii* PCC 9212. Red boxes represent genes encoding core subunits of PS I; green boxes represent genes encoding core subunits of PS II; blue boxes represent genes encoding core components of the phycobilisome; brown boxes represent regulatory *rfp* genes; and gray boxes represent genes that are not found in other FaRLiP clusters. **(B)** Schematic showing deletion of *rfpA*. The small arrows (P1 and P2) indicate the positions of the primers used for PCR verification of deletion. **(C)** Schematic showing deletion of *rfpB*. The small arrows (P3 and P4) indicate the positions of primers used for PCR verification of the deletion. **(D)** Schematic showing deletion of *rfpC*. The small arrows (P5 and P6) indicate the positions of primers used for PCR verification of the deletion. **(E)** Agarose gel electrophoresis of amplicons showing complete segregation of wild-type and mutant *rfpA* alleles. **(F)** Agarose gel electrophoresis of amplicons showing complete segregation of wild-type and mutant *rfpB* alleles. **(G)** Agarose gel electrophoresis of amplicons showing complete segregation of wild-type and mutant *rfpC* alleles.

The authors apologize for this error and state that this does not change the scientific conclusions of the article in any way. The original article has been updated.

