# Supplementary material for: RfpA, RfpB, and RfpC are the Master Control Elements of Far-Red Light Photoacclimation (FaRLiP)
Source: Front Microbiol. 2015 Nov 25;6:1303. doi: 10.3389/fmicb.2015.01303 (PMC4658448; doi:10.3389/fmicb.2015.01303)
Supplement: Supplementary file 1 [file Table1.PDF]

Supporting Information

Supplemental Tables S1

Legend for Table S2

RfpA, RfpB and RfpC are the master control elements of far-red light photoacclimation (FaRLiP)

Chi Zhao[1], Fei Gan[1], Gaozhong Shen[1], and Donald A. Bryant[1,2]*

[1]Department of Biochemistry and Molecular Biology, The Pennsylvania State University, University Park, PA 16802 USA; [2]Department of Chemistry and Biochemistry, Montana State University, Bozeman, MT 59717 USA.

***Correspondence:** Dr. Donald A. Bryant, 403C Althouse Laboratory, Department of Biochemistry and Molecular Biology, The Pennsylvania State University, University Park, PA 16802 USA. Phone; 814-865-1992; Fax: 814-863-7024; E-mail: dab14@psu.edu

Running Title: Master control elements of far-red light photoacclimation

Table S1. Sequences of primers used for: 1) generation of constructs for deletion of the *rfpA*, *rfpB* and *rfpC* genes in *Chlorogloeopsis fritschii* PCC 9212*,* and of the *rfpA* and *rfpB* genes in *Chroococcidiopsis thermalis* PCC 7203; 2) PCR analysis in segregation verification for transconjugants; and 3) gene expression comparison by RT-PCR for selected genes in wild type and mutants of *Chlorogloeopsis fritschii* PCC 9212. Restriction sites are underlined.

Em-1 (SacI)	5'-GCGCGAGCTCGCGTGCTATAATTATACTAATTTTATAAGGAGGAAAAAAT-3'
Em-2 (SphI)	5'-TTAAGCATGCTGAGTGAGCTGATACCGCTCGCCGCAG-3'
9212 *rfpA* up-1 (*XhoI*)	5'-TTAACTCGAGCTAACACGCTGCCATATCAAGTGCCAT-3'
9212 *rfpA* up-2 (*SacI*)	5'-GGTTGAGCTCCATCATCTAAAACATCATGGCAAAGT-3'
9212 *rfpA* down-1 (*SphI*)	5'-GGAAGCATGCTAGCACCTTCTATTTTACGTTACCTATTAGTTAG-3'
9212 *rfpA* down-2 (BamHI)	5'-CTCTGGATCCGTTCGTTAGGGGATTAACTATTAGCT-3'
9212 *rfpB* up-1 (*XhoI*)	5'-TTAACTCGAGCAGGCTGATGAAATTGCAAGTAGTCAAAGT-3'
9212 *rfpB* up-2 (*SacI*)	5'-TTAAGAGCTCCTAAAATGTCGCTGCGGCTAAAAACTTGAT-3'
9212 *rfpB* down-1 (*SphI*)	5'-TTAAGCATGCCAAAGGCGTTAAATGACACTAGCAAT-3'
9212 *rfpB* down-2 (*BamHI*)	5'-TTAAGGATCCGTCAACCCATCCATATCTGGCAT-3'
9212 *rfpC* up-1 (*XhoI*)	5'-TTAACTCGAGCGCGTTTGGCTTTTGAAGTTGAGCAGAT-3'
9212 *rfpC* up-2 (*SacI*)	5'-GGTTGAGCTCCAAAATGCGTTTTGTCATTTGTCATTAGTTATTAGT-3'
9212 *rfpC* down-1 (*SphI*)	5'-CCTTGCATGCGATAGGTCAGTGTGAATATATTTCAGCCAT-3'
9212 *rfpC* down-2 (*BamHI*)	5'-TTAAGGATCCTGCCATCACTAAACCACCAATTGCAGT-3'
7203 *rfpA* up-1 (*XhoI*)	5'-TTAACTCGAGCTAACACGCTGCCATATCAAGTGCCAT-3'
7203 *rfpA* up-2 (*SacI*)	5'-GGTTGAGCTCCATCATCTAAAACATCATGGCAAAGT-3'

7203 *rfpA* down-1 (*SphI*)	5'-GGAAGCATGCTAGCACCTTCTATTTTACGTTACCTATTAGTTAG-3'
7203 *rfpA* down-2 (*BamHI*)	5'-CTCTGGATCCGTTCGTTAGGGGATTAACTATTAGCT-3'
7203 *rfpB* up-1 (*XhoI*)	5'-TTAACTCGAGCAGGCTGATGAAATTGCAAGTAGTCAAAGT-3'
7203 *rfpB* up-2 (*SacI*)	5'-TTAAGAGCTCCTAAAATGTCGCTGCGGCTAAAAACTTGAT-3'
7203 *rfpB* down-1 (*SphI*)	5'-TTAAGCATGCCAAAGGCGTTAAATGACACTAGCAAT-3'
7203 *rfpB* down-2 (*BamHI*)	5'-TTAAGGATCCGTCAACCCATCCATATCTGGCAT-3'
P1: 9212 *rfpA*-1	5'-CATAATGGTATACAACAAACAAAAGCAGCGTTAACT-3'
P2: 9212 *rfpA*-2	5'-GAGGATTTTTCTGGAGGTTTTGCAAGGTTGT-3'
P3: 9212 *rfpB*-1	5'-GTAGTCCTAGCTTTTCCAAAGATTGTGGAGCGAT-3'
P4: 9212 *rfpB*-2	5'-CATGACAAGAATCTACTGCTCGAAACCGAAT-3'
P5: 9212 *rfpC*-1	5'-GTTATTCCAAATTCAAGATCAAGGGCGAGGT-3'
P6: 9212 *rfpC*-2	5'-GCTATATTGGGCACCACGTCTATTGCAGAAACT-3'
P7: 7203 *rfpA*-1	5'-CATTCAGCTCAACACGTTGGAAGAACCAT-3'
P8: 7203 *rfpA*-2	5'-GTAAAATGACAGGAATATTTTGCGTCAAAGGAT-3'
P9: 7203 *rfpB*-1	5'-GATCGCCCTTACTGTCAGATGCTTGCAACT-3'
P10: 7203 *rfpB*-2	5'-GTTGTCCCAGTAAAGATGGTAAACGTTCTTCCT-3'
9212 16S RT-1	5'-GATACTAGGCGTTGCGTGTATCGACCCAC-3'
9212 16S RT-2	5'-GTTGCAGCCTGCGATCTGAACTGAG-3'
9212 *psaA1* RT-1	5'-CAAAGACTTGTTGACCGAGCTGTATCCTAGC-3'
9212 *psaA1* RT-2	5'-TGAAGATGGCAGCGTGAGCAGCACC-3'
9212 *psbB1* RT-1	5'-GTTGGCGATCCCAATTACAATCCAGGTTTC-3'
9212 *psbB1* RT-2	5'-AAGAATACAGCAGCAATACTGCTAGAAAGCACGG-3'
9212 *cpcB* RT-1	5'-ATGTTAGACGCATTTGCCAAGGTGGTTTC-3'
9212 *cpcB* RT-2	5'-GCGCGATCGAAGTAGCTAGCCAATTCAGAC-3'
9212 *psaA2* RT-1	5'-TCGTACCAATTGGGGTATCGGTCACAGCAT-3'
9212 *psaA2* RT-2	5'-TATCTTGAGGACGACCAAAAGCCCGCAT-3'
9212 *psaB2* RT-1	5'-CAAGGTGCAGGTACAGCAATTCTCACATTC-3'
9212 *psaB2* RT-2	5'-CGCTCTAAGACATTACCTTTATTCTGTTCTGGGTC-3'
9212 *psbA4* RT-1	5'-CAAGTGCCATAAATAAACTTGGGCAGACCT-3'
9212 *psbA4* RT-2	5'-ATCCTATTTGGGAAGCAGCTTCCATTGATGAG-3'
9212 *psbA5* RT-1	5'-GTAACCATGGGCTGCAACAATGTTATAGGT-3'
9212 *psbA5* RT-2	5'-CGATTGGATTGCACTTTTACCCAATTTG-3'
9212 *apcE2* RT-1	5'-GTCCACCCTTAACTTGGGATTCCAATTCAT-3'
9212 *apcE2* RT-2	5'-GCCGACGAGAATGAAACGGTCTATGCG-3'

Table S2

Complete RNAseq data for *Chl. fritschii* 9212 grown in WL (T=0) and grown in FRL for 12, 24, 48, and 336 hours (2 weeks; designated as “FR” in the table). Ratios of transcripts were calculated relative to the WL (T=0) sample for all samples from cells grown in FRL and ratios were also calculated for the 48-h sample relative to the FR sample (336 h, two weeks).
